# *In Vitro* Drug Release Kinetics and Antioxidant Activity of Metformin-Loaded Niosomes

**DOI:** 10.1021/acsomega.5c03679

**Published:** 2025-11-24

**Authors:** Emine Esin Çalışkan, Yalçın Çelik Aydın, Emine Nur Ozbek, Ilayda Alcın, Emrah Kilinc, Gunay Yetik Anacak, Emel Oyku Cetin Uyanikgil

**Affiliations:** a Faculty of Pharmacy, Department of Biopharmaceutics and Pharmacokinetics, 37509 Ege University, Izmir 35040, Turkey; b Gediz Vocational School of Health Services, Department of Pharmacy Services, Kütahya Health Sciences University, Kütahya 43020, Turkey; c Faculty of Pharmacy, Department of Pharmacology, 37509 Ege University, Izmir 35040, Turkey; d Faculty of Pharmacy, Department of Analytical Chemistry, 37509 Ege University, Izmir 35040, Turkey; e Faculty of Pharmacy, Department of Pharmacology, Acibadem Mehmet Ali Aydinlar University, Istanbul 34752, Turkey

## Abstract

Metformin is an antidiabetic drug that is widely used in the treatment of type 2 diabetes mellitus (T2DM) and is known to reduce oxidative stress. Drug-loaded niosomes enhance the cellular uptake of drugs, resulting in improved antioxidant effects. In this study, we formulated metformin-loaded niosomes, aiming to enhance cellular drug uptake and augment antioxidant effects. The particle size, polydispersity index, and zeta potential values were found to be 153.8 nm, 0.449, and −9.32 mV respectively. Morphological observations conducted through scanning electron microscopy (SEM) provided insights into the distinctive structure of the niosomes. Entrapment efficiency of drug-loaded niosomes was determined to be 68%. In vitro drug release studies, performed by using the dialysis bag method, exhibited a release profile consistent with Hixson–Crowell kinetics. After characterization of the formulations, the antioxidant activity of metformin-loaded niosomes on Pyrogallol-induced reactive oxygen species (ROS) formation in the mouse brain was compared with metformin treatment alone. ROS formation was measured by a luminol–lucigenin chemiluminescence assay. Metformin-loaded niosomes significantly reduced ROS formation compared to metformin treatment alone. Metformin-loaded niosomes offer potential for enhanced antioxidant effects and bioavailability by increasing cellular uptake of metformin.

## Graphical abstract

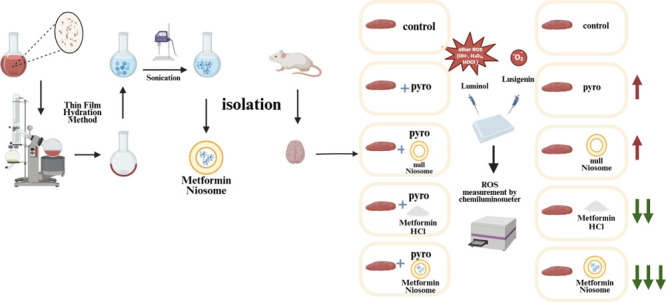

## 1 Introduction

The disadvantages of conventional drug delivery systems, such as limited pharmacokinetic performance and tissue distribution, adverse effects, clearance by the reticuloendothelial system, and inadequate cellular uptake, have necessitated the development of next-generation drug delivery technologies.^1,2^ In recent years, nanostructured drug delivery systems have emerged as promising solutions to these challenges. Among them are various carrier platforms, including polymeric nanoparticles, liposomes, niosomes, micelles, dendrimers, and mesoporous silica nanoparticles.^3^ Liposomes and niosomes are particularly notable for their ability to enhance bioavailability, enable targeted delivery, and minimize toxic effects.^4^

Niosomes are multilamellar vesicular drug delivery systems structurally similar to liposomes, containing both hydrophilic and hydrophobic groups.^1^ While both can encapsulate hydrophilic and lipophilic drugs and offer targeted delivery with improved bioavailability,^5,6^ niosomes differ from liposomes in that they are composed of nonionic surfactants such as Tween, Span, and Brij rather than phospholipids.^7^ Due to this structural difference, niosomes are more resistant to oxidation, cost-effective and stable compared to liposomes.^7,8^

Common methods for preparing niosomes include thin-film hydration, ether injection, reversed-phase evaporation, and sonication. In the frequently used thin-film hydration method, cholesterol and nonionic surfactants are dissolved in the organic phase in a round-bottomed flask. The organic phase is then evaporated under low pressure in a rotary evaporator to obtain a thin-film layer. The film is then hydrated with water or phosphate buffer to form multilamellar vesicles.^7,9,10^

Metformin is a widely prescribed first-line drug for type 2 diabetes mellitus (T2DM), with several benefits beyond its glucose-lowering effect, including neuroprotective and antioxidant properties. Recent studies suggest that metformin may be a potential therapeutic agent in neurodegenerative diseases such as Alzheimer’s disease,^11^ Parkinson’s disease,^12^ Huntington’s disease,^13^ and major depressive disorders.^14^

Given the brain’s high metabolic activity and relatively low levels of endogenous antioxidant enzymes, it is particularly vulnerable to oxidative damage caused by reactive oxygen species (ROS).^15^ Oxidative stress is also recognized as a key factor in the pathogenesis of major depressive disorder.^16^ Therefore, metformin has been proposed as a potential therapeutic agent in diabetes-induced depression due to its known neuroprotective and antioxidant effects.

In recent years, several metformin-loaded nanosystems have been developed and evaluated, including solid lipid nanoparticles,^17^ mesoporous silica nanoparticles,^18^ microspheres,^19^ and chitosan-silver nanoparticles.^20^ The novel aspect of this research lies in the investigation of the antioxidant activity of metformin-loaded niosomes in brain tissue, specifically their ability to reduce ROS formation and demonstrate superior antioxidant efficacy compared to metformin alone.

## 2 Materials and Methods

### 2.1 Materials

Sigma-Aldrich provided Span 60, Tween 60, and cholesterol. All chemicals and solvents were provided by Merck and were of high-performance liquid chromatography (HPLC) or analytical grade, and no additional purification was performed.

### 2.2 Preparation of Niosome Formulations

Unloaded and metformin-loaded niosome formulations were prepared via the film hydration method (Figure 1).^21^ The amount of surfactants in the formulation was determined by calculating the HLB value to be 8.6. Briefly, cholesterol (38.66 mg) and surfactants [Span 60 (26.6 mg) and Tween 60 (50.16 mg)] [1:1 molar ratio] were weighed and dissolved in chloroform (10 mL) in a round-bottom flask. The chloroform was evaporated at 60 °C under reduced pressure using a rotary flash evaporator (IKA RV 8 V, Turkey). The thin films were hydrated with 10 mg of metformin and 10 mL of phosphate buffered saline (PBS), pH 7.4, and the flask was kept rotating at 60 °C and 100 rpm (rpms). Formulations were sonicated for three cycles, 35% power in a prob sonicator for 15 min (Qsonica LLC, Newton, Connecticut). Vesicle suspensions were centrifuged (Optima XE-90, Beckman Coulter, Brea, California) at 20,000 rpm for 60 min at 4 °C. Finally, for lyophilization, 1 mL of 3% trehalose solution was added to the washed niosome formulation, and the system was frozen at −20 °C and lyophilized using a LaboGene ScanVac CoolSafe freeze-dryer device (Scandinavia) for 48 h.

**Figure 1:**
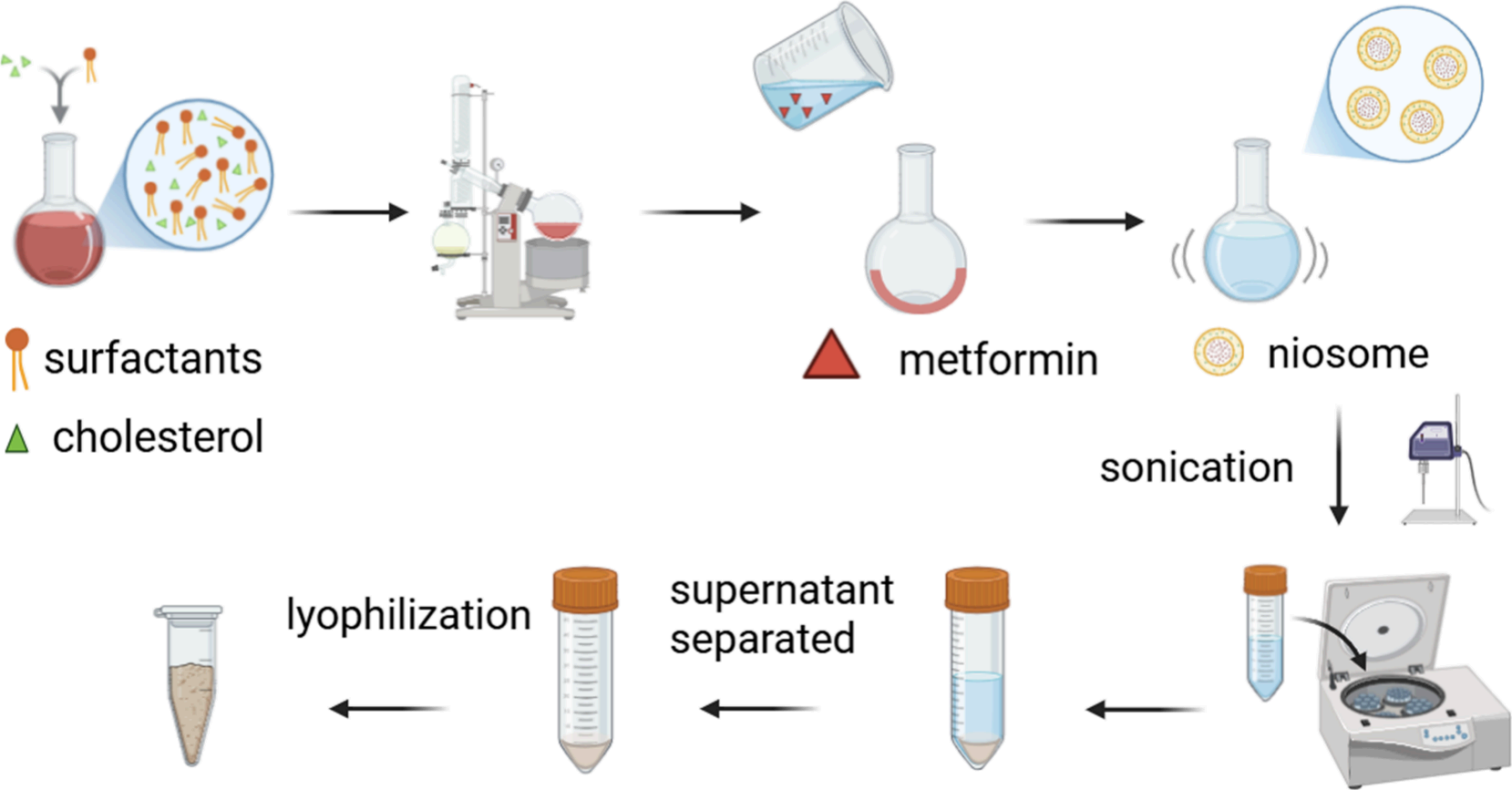
Niosome formulation preparation with a thin-film hydration method

### 2.3 Characterization of Niosomes

#### 2.3.1 Determination of Entrapment Efficacy in Vesicles

Metformin-loaded niosome (Figure 2) formulations were centrifuged at 20,000 rpm for 60 min at 4 °C using a refrigerated centrifuge to separate niosomes from nonentrapped drug. The concentration of the free drug in the supernatant was determined by HPLC.

**Figure 2:**
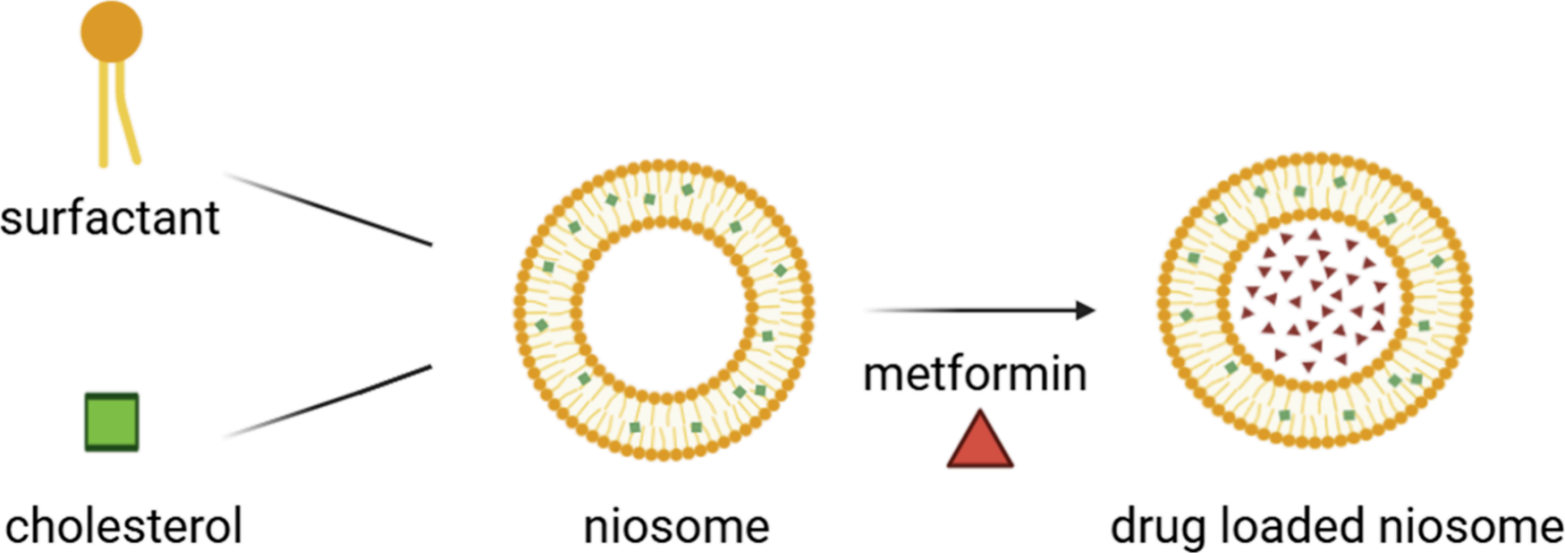
Formation of metformin-loaded niosomes.

The samples were evaluated with a validated HPLC method (Thermo Dionex UltiMate 3000) equipped with a PDA detector (DAD-3000) detector set at 281 nm, using a C18 column (250 mm × 4.6 mm–5 mcm); Thermo ODS Hypersil). The column temperature was 40 C. The mobile phase consisting of 0.01 M H_3_PO_4_:acetonitrile:methanol (55:35:10) was fluxed at 2.5 mL/min. UV/vis spectra were collected in 200–400 nm, while absorbance was monitored in four different channels at 228, 232, 241, 280, and 320 nm wavelengths. This process was repeated three times to ensure that the free drug was completely removed. The percentage of drug entrapment in niosomes was calculated.^22^

#### 2.3.2 DSC and FTIR Analyses

The thermal properties of the obtained niosome formulations were analyzed by using DSC (PerkinElmer DSC 8000). Approximately 5 mg of the prepared niosomes was placed into standard aluminum sample molds, and the molds were emptied by providing 5 mL of nitrogen per minute. This process was carried out by heating at 10 °C/min between 20 and 300 °C. DSC curves were drawn.

The FTIR spectra of the formulations were obtained using a JASCO FTIR-4200 spectrometer with KBr pellets for solid specimens.

#### 2.3.3 Scanning Electron Microscopic Analysis

The prepared niosome formulations were attached to double-sided adhesive tape on a metal grid and coated with gold using a sputter coater using a vacuum evaporator (K550X Sputter Coater, Emitech) at a thickness of 10 nm for 1.5 min under conditions of 15 mA and 6 × 10 ^–2^ mbar. They were then examined and photographed with a SEM device (Philips XL-30S FEG) under conditions of 100,000× magnification and 7.5 kV at room temperature. The morphological properties of null and metformin-loaded niosomes were investigated.

#### 2.3.4 Particle Size, Zeta Potential, and Polydispersity Index Measurement

The mean particle size and distribution of niosome formulations were determined with dynamic light scattering (DLS) using the Malvern Zetasizer Nano ZS90 (Malvern Instruments, Malvern, U.K.) at 25 °C and 120 s equilibration time. Null and metformin-loaded niosome formulations were diluted with ultrapure water (1:400) before analysis. The particle size and PDI value measurements were carried out six times, and the results were presented as the mean (nm). The zeta potential value of niosome formulations was determined using the Malvern Zetasizer Nano ZS90 (Malvern Instruments) with a folded capillary cell (DTS1070). Before analysis, the niosome formulations were diluted with ultrapure water (1:400). Zeta potential measurements were carried out six times, and the results were presented as zeta potential (mV).^23^

### 2.4 Spectrophotometric Analysis

A Shimadzu UV-1280 UV/vis spectrophotometer was employed for entire spectroscopic acquisitions using a standard quartz cell with a 1 cm light path. For the preparation of metformin stock solution (MetSS), 2.5 mg of metformin was weighed properly and immediately transferred to a 25 mL volumetric flask where it was dissolved in 10 mL of pH 7.4 10 mM phosphate buffer solution (PBS) under 5 min sonication. Finally, the volume of the flask was made up to its mark with the same solvent to obtain the final concentration (100 μg/mL) of the component. Diluted standard calibration solutions with a concentration range of 1 to 25 μg/mL were prepared by placing 10, 50, 100, and 250 μL of MetSS into an Eppendorf tube and completing the final volume to 1 mL with PBS. 10 μg/mL standard solution of metformin was scanned in the wavelength range of 200–600 nm, and the λ_max_ was determined to be 232 nm in PBS. The absorbance of all standard solutions was acquired at 232 nm, and the calibration curve was plotted.

### 2.5 *In Vitro* Dissolution Studies

*In vitro* dissolution studies were carried out in a shaking incubator at 100 rpm and 37 °C using a dialysis membrane method in a release medium containing pH 7.4 phosphate buffer.^24^ Dialysis membranes with a pore diameter of 12–14 kDa were washed in distilled water for 15 min, and then 1 mL of niosome formulation was added to each side of the membrane and sealed. The studies were conducted in triplicate for metformin-loaded niosome formulations at 30 min, 1 h, 2 h, 3 h, 4 h, 6 h, 8 h, 10 h, 12 h, and 24 h. At each time point, 1 mL of the sample was taken from the release medium and replaced with 1 mL of phosphate buffer. The obtained fractions were analyzed by UV–vis molecular absorbance spectroscopy, and the release graphs of the formulations were drawn.

### 2.6 Release Kinetics Modeling

Cumulative drug release data were plotted against time, and the suitability of different kinetic models was evaluated. For this purpose, the experimental data were fitted to several widely accepted mathematical models: zero-order, first-order, Higuchi, Hixson–Crowell, and Korsmeyer–Peppas.

The zero-order model is described by the following equation:

$$Qt=Q_{0}+K_{0}t$$ 1

where *Q_t_* is the amount of drug released at time *t*, *Q*_0_ is the initial amount of drug (often taken as zero), and *K*_0_ is the zero-order release rate constant.

The first-order model is represented by the following logarithmic equation:

$$\log\;C=\log\;C_{0}-K_{t}/(2.303)$$ 2

where *C*_0_ is the initial drug concentration, *K* is the first-order release rate constant, and *t* is thetime with the slope of the curve being −K/2.303.

The Higuchi model equation is

$$Q=K_{H}\times t^{1/2}$$ 3

where *Q* is the cumulative amount of drug released at time *t* and *K*_H_ is the Higuchi release constant.

The Hixson–Crowell model is expressed as

$${W_{0}}^{1/3}-{W_{t}}^{1/3}=\kappa \times t$$ 4

where *W*_0_ is the initial amount of drug in the dosage form, *W_t_* is the amount remaining at time *t*, and κ is a constant incorporating surface area-to-volume considerations.

The Korsmeyer–Peppas model is given as

$$M_{t}/M_{\infty }=K\times t^{n}$$ 5

where *M_t_*/*M*_∞_ is the fraction of drug released at time *t*, *K* is the kinetic constant, and *n* is the release exponent that characterizes the mechanism of drug release. The value of *n* provides insight into the release mechanism (Table 1).

**Table 1:** Korsmeyer–Peppas Mode Drug Release Mechanism

*n* ≤ 0.45	Fickian diffusion (diffusion-controlled release)
0.45 < *n* < 0.89	anomalous (non-Fickian) transport (combination of diffusion and polymer relaxation)
*n* = 0.89	case II transport (polymer relaxation-controlled release)
*n* > 0.89	super case II transport (swelling and relaxation-dominant release)

### 2.7 Measurement of Reactive Oxygen Species (ROS) Formation by Chemiluminescence

ROS formation was detected using the lucigenin and luminol chemiluminescence assay, following the modified protocol described by Münzel et al.^25,26^ The study was approved by the Animal Experiment Local Ethical Committee of Ege University (No. 2019-098) in agreement with European guidelines for animal care. The animals (25–30 g male Swiss albino mice) were housed at 20–25 °C with 50–60% humidity under a 12 h light–dark cycle with free access to food and water. On the day of the experiment, animals were sacrificed by cervical dislocation under ketamine (60 mg/kg)/xylazine (5 mg/kg) anesthesia. Brain tissues were isolated and incubated with pharmacological agents (1 mM metformin alone, null niosomes, or 1 mM metformin-loaded niosomes) in HEPES-buffer (pH 7.4). Pyrogallol (Pyro, 100 μM) was used as an oxidant agent to induce ROS formation. After treatments, lucigenin or luminol (5 μM) was added and ROS formation was measured luminometrically using Varioskan Flash multimode reader (Thermo Scientific, USA). Counts were recorded at 1 min intervals over a 5 min period, and results were expressed as the area under the curve (AUC) of relative light units (rlu) per milligram of wet tissue.^26^

### 2.8 Statistical Analysis

All statistical analyses and graphical representations were performed using GraphPad Prism software, version 8 (GraphPad Software, San Diego, California, USA). Data are presented as mean ± standard error of the mean (SEM). Statistical significance was defined as *p* < 0.05. Statistical differences between groups were assessed using One-way analysis of variance (ANOVA) followed by Bonferroni’s post hoc multiple comparison test. *n* indicates the number of animals included in each group.

## 3 Results and Discussion

### 3.1 Characterization of Niosomes

The encapsulation efficiency of niosomes reflects the loading capacity of vesicles for the use as therapeutic agents. Encapsulation efficiency is mainly dependent on the type of nonionic surfactant, synthesis method, and other substances used in the formulation process such as cholesterol.^27^ Studies have revealed that the hydrophilic–lipophilic balance (HLB) value is a significant factor affecting encapsulation efficiency, with an optimum HLB value of 8.6 identified for maximum drug loading capacity.^28^ The loading efficiency of niosomes was calculated based on the difference between the initial drug concentration and the amount of drug not encapsulated in the medium. Accordingly, the encapsulation efficiency of the niosome formulation was found to be 68% for metformin, demonstrating the effective loading of the drug within the vesicles. This result is consistent with findings in the literature, indicating the suitability of the selected formulation method for achieving high drug entrapment.^29^

As the next step of the characterization processes, the thermal behaviors of metformin, null niosome, and metformin-loaded niosome formulation were evaluated by differential scanning calorimetry (DSC) analysis. In Figure 3, the DSC curve of metformin exhibited an endothermic peak at approximately 228 °C; this corresponds to the melting point of the drug, which indicates its crystal structure and is consistent with the literature. In contrast, the null niosome exhibited a broad transition at around 48 °C, which is characteristic of the lipid components used in its formulation. For the metformin-loaded niosome, the absence of the sharp endothermic peak at 228 °C suggests that metformin may be molecularly dispersed or encapsulated within the niosomal structure, resulting in a loss of its crystalline properties. These results consistent with the successful incorporation of metformin into the niosomal carrier system.^30,31^

**Figure 3:**
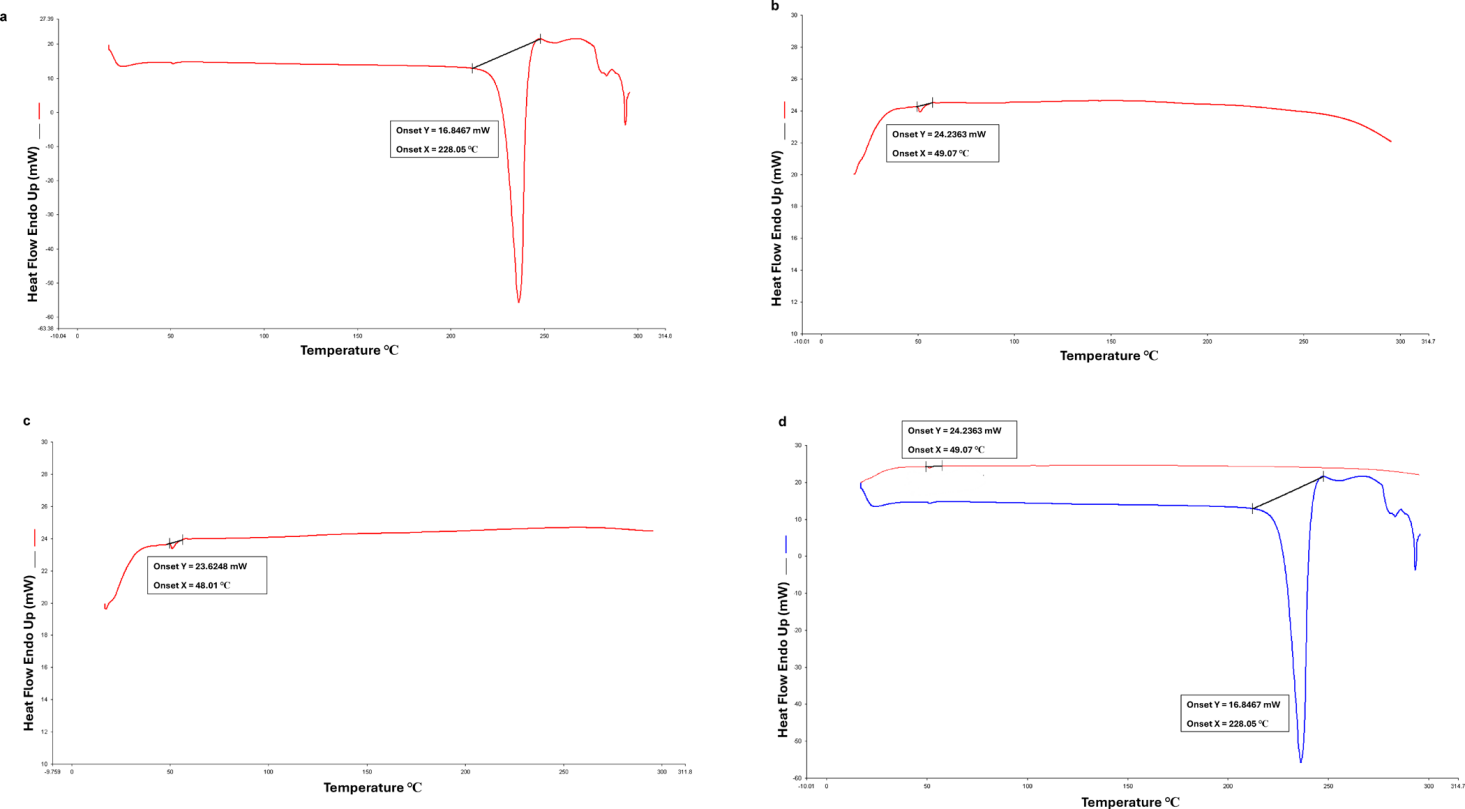
DSC curves of (a) metformin, (b) null niosome, (c) metformin niosome, and (d) metformin + metformin-loaded noisome formulations.

Characterization of the metformin-loaded niosome formulation was continued with FTIR analyses. DSC results showed a change in the thermal behavior of the metformin–niosome compared with the metformin and null niosome.

The FTIR spectra of pure cholesterol (Figure 4), metformin (Figure 5), null niosomes (Figure 6) and metformin-loaded niosomes (Figure 7) were compared to evaluate possible interactions and confirm drug encapsulation. Metformin exhibited characteristic peaks at approximately 3360 cm^–^^1^ (N–H stretching), 1627 cm^–^^1^ (C=N stretching), 1580 cm^–^^1^ (N–H bending), and 1060 cm^–^^1^ (C–N stretching).

**Figure 4:**
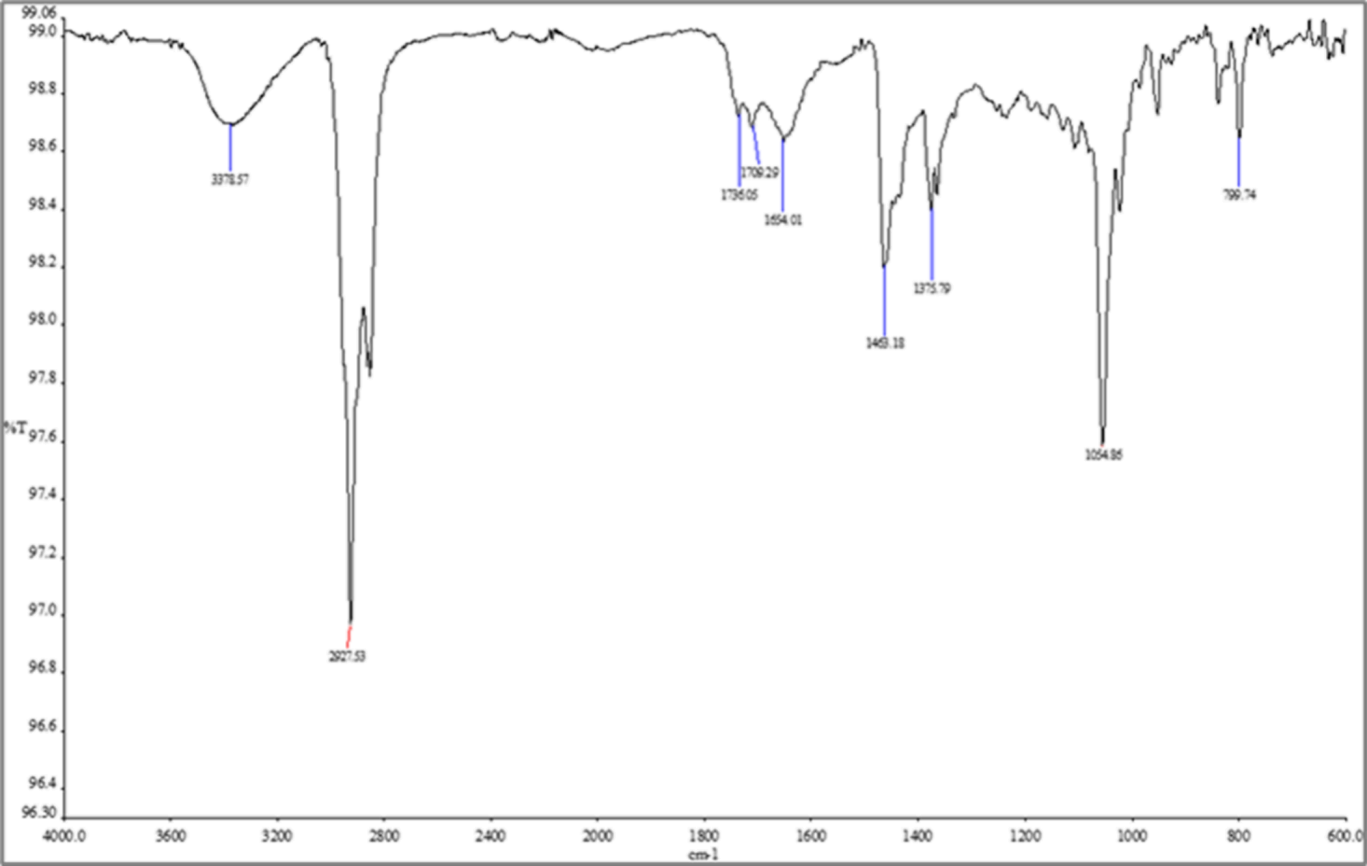
FTIR spectra of cholesterol. Spectra are presented as % transmittance (%T) versus wavenumber (cm^–^^1^).

**Figure 5:**
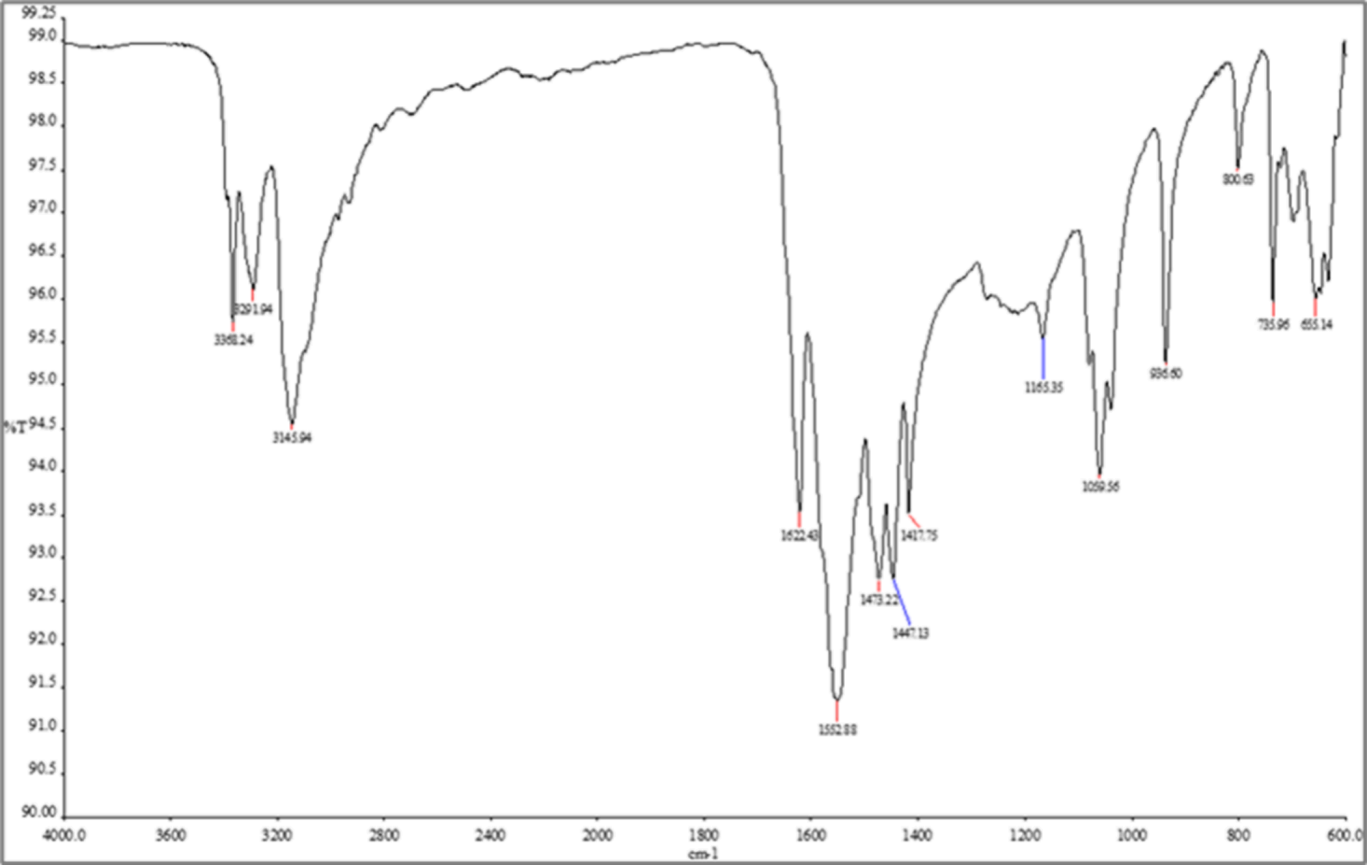
FTIR spectra of metformin. Spectra are presented as % transmittance (%*T*) versus wavenumber (cm^–^^1^).

**Figure 6:**
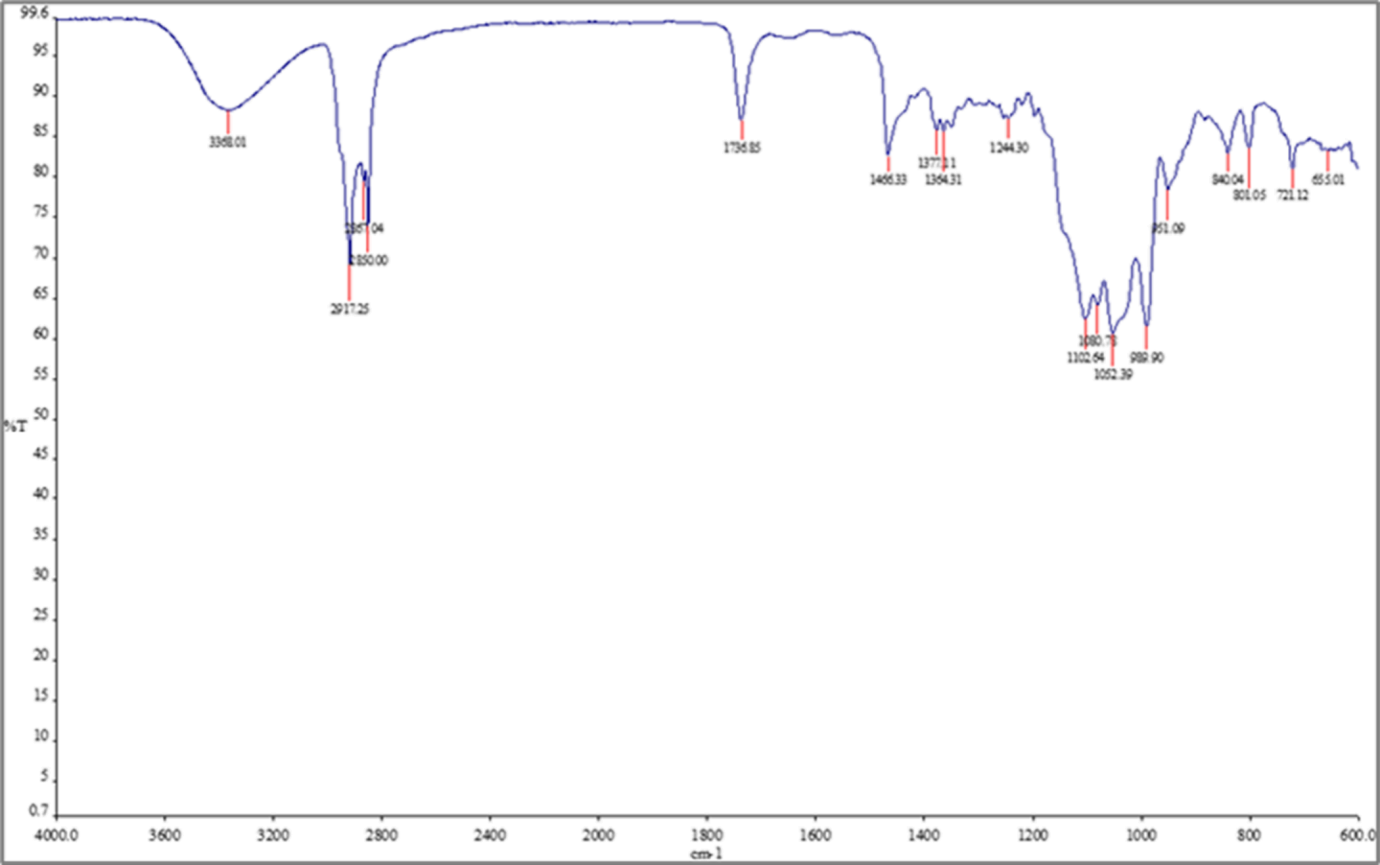
FTIR spectra of the null niosome. Spectra are presented as % transmittance (%*T*) versus wavenumber (cm^–^^1^).

**Figure 7:**
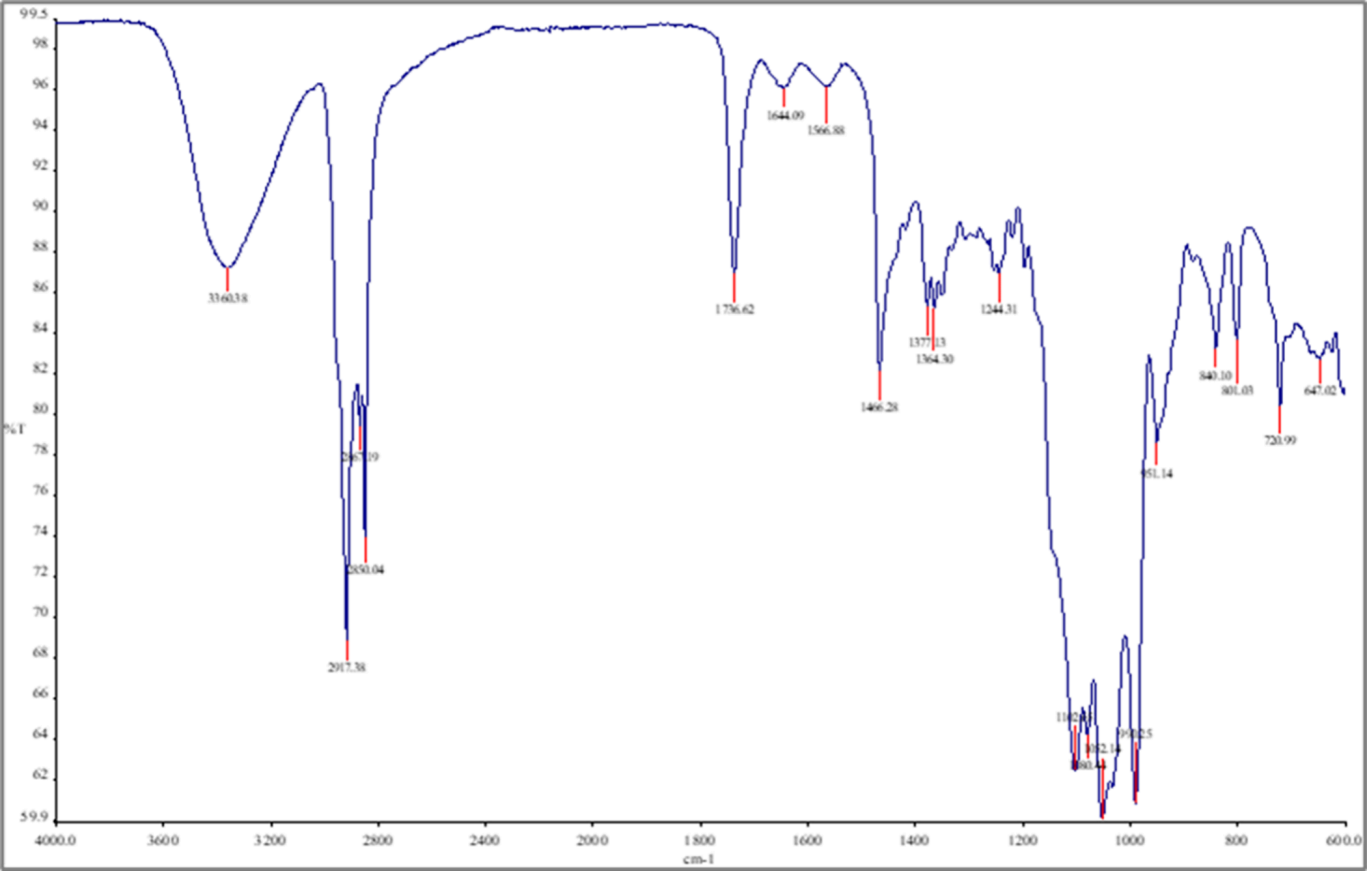
FTIR spectra of the metformin niosome. Spectra are presented as % transmittance (%*T*) versus wavenumber (cm^–^^1^).

In the metformin-loaded niosome spectrum, some of these peaks were shifted or broadened and their intensity slightly reduced, compared to the pure drug spectrum. This suggests possible intermolecular interactions between metformin and the surfactant/cholesterol bilayer. For instance, the N–H stretching band at 3360 cm^–^^1^ was slightly shifted and overlapped with the broad peak of the niosomal components.

The blank niosomes and cholesterol spectra, as expected, did not show metformin-specific peaks. These observations, though qualitative, are indicative of drug entrapment and possible hydrogen bonding or hydrophobic interactions within the niosomal structure.

Collectively, these findings are confirmed by both thermal and structural evidence, supporting the successful loading and encapsulation of metformin into the niosomal carrier system. The findings related to are consistent with the literature.^32,33^

In SEM images in Figure 8, niosomes were observed in a uniform spherical structure, and their particle sizes were consistent with DLS results. No difference in surface structure was observed between metformin-loaded and empty niosomes. The reason for this is that metformin is a water-soluble compound and is present in the aqueous core and there is no difference in surface.^34^

**Figure 8:**
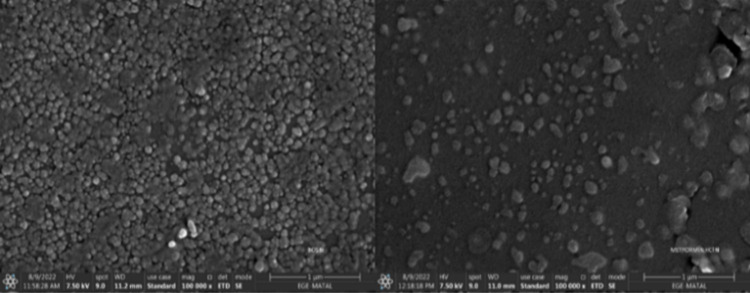
SEM images of null noisome and metformin-loaded niosome, respectively.

Particle size in nanostructured drug delivery systems is a crucial physical attribute that affects stability, encapsulation efficiency, drug release profile, biodistribution, mucoadhesion, and cellular uptake. Targeting tissues with high blood flow such as the brain and tumor tissues require particle sizes in the range of 5–200 nm.^22^ The particle sizes of empty (Figure 9) and metformin-loaded niosome (Figure 10) formulations were found to be 141.5 ± 3.2 and 153.8 ± 4.0 nm, respectively. The obtained results fall within the commonly reported optimal size range (typically below 200 nm) for facilitating transport across biological barriers, including the blood–brain barrier.

**Figure 9:**
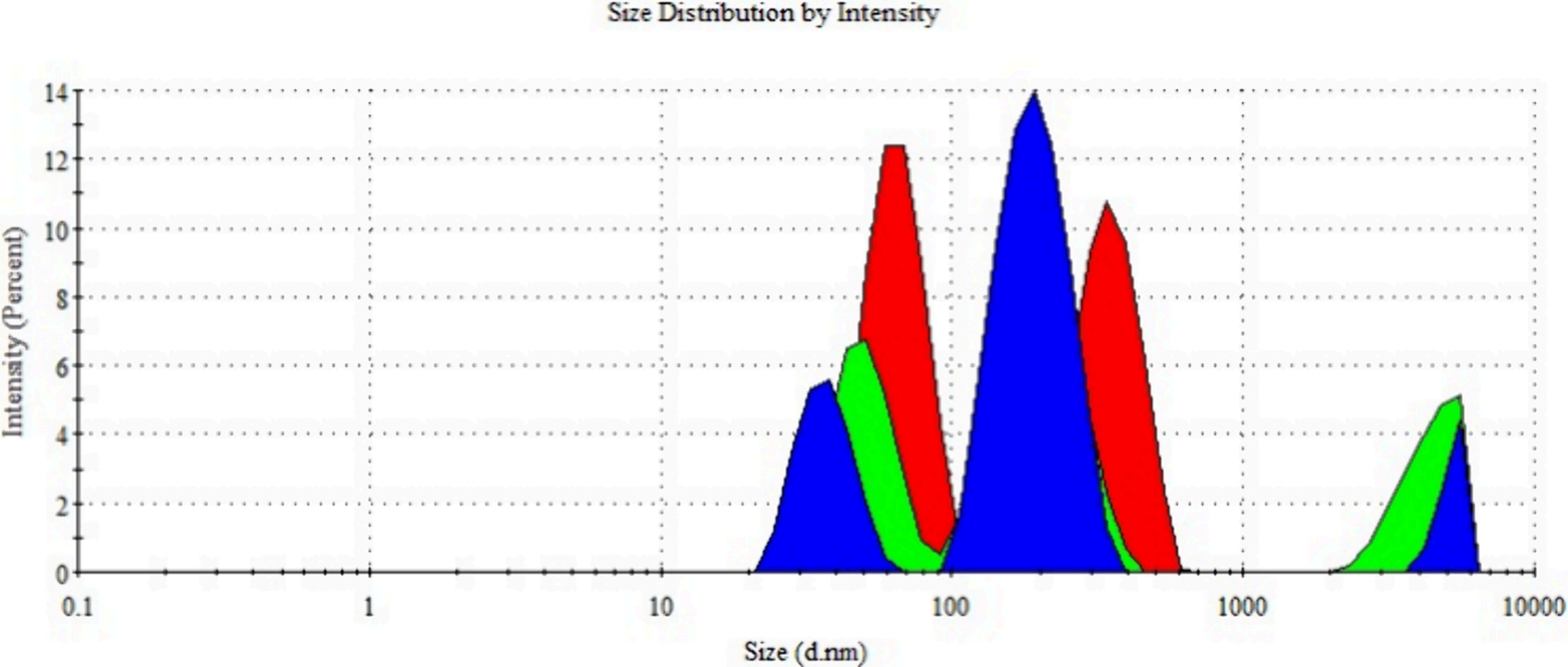
Size distribution of null-niosome formulations.

**Figure 10:**
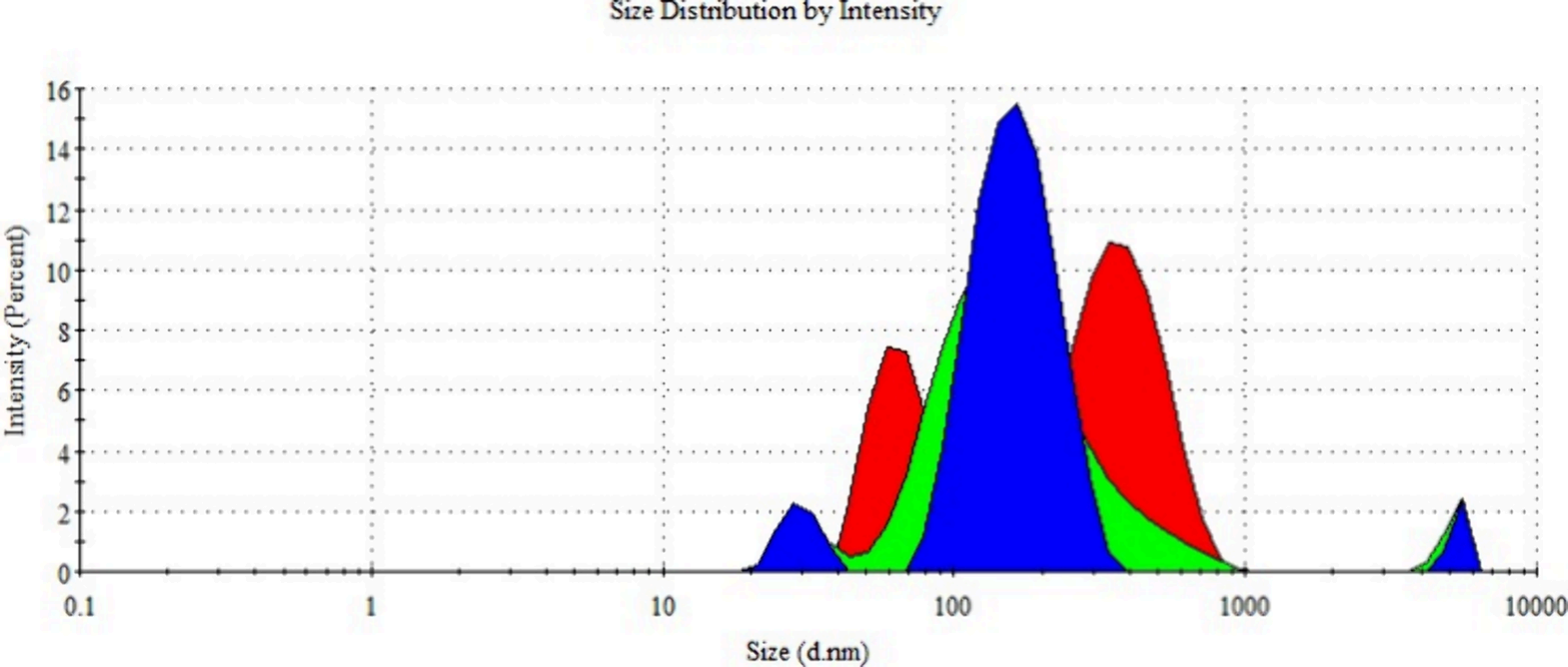
Size distribution of metformin-loaded niosome formulations.

The polydispersity index (PDI) is an important parameter indicating particle uniformity and stability in particulate systems. This value ranges from 0.0 to 1.0, with 0.0 indicating maximum uniformity of particles.^35^ 0.3 is considered an ideal PDI value for niosome formulations.^35^ The measured PDI values using the dynamic light scattering (DLS) technique for empty and metformin-loaded formulations were 0.395 ± 0.03 and 0.449 ± 0.04, respectively, indicating satisfactory uniformity.

Zeta potential is a parameter indicating stability in colloidal systems. Particles with zeta potential values below −30 or above +30 mV are considered stable.^36^ Particles within this range are prone to aggregation. The measured zeta potential values for empty (Figure 11) and metformin-loaded (Figure 12) niosomes using the DLS technique were −8.22 ± 0.06 and −9.32 ± 0.8 mV, respectively, suggesting a tendency for aggregation based on the obtained PDI and zeta potential values.

**Figure 11:**
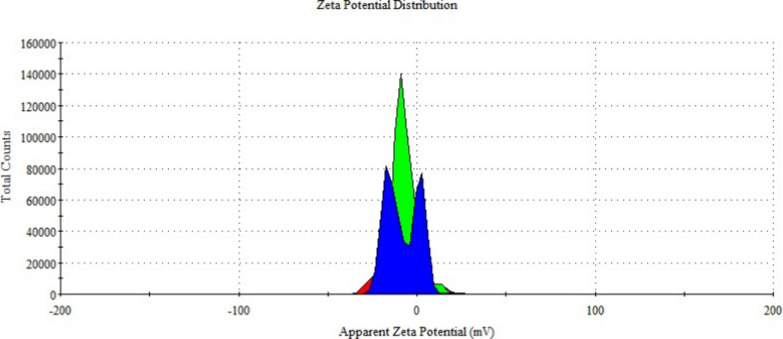
Zeta potential distribution of null niosome formulations.

**Figure 12:**
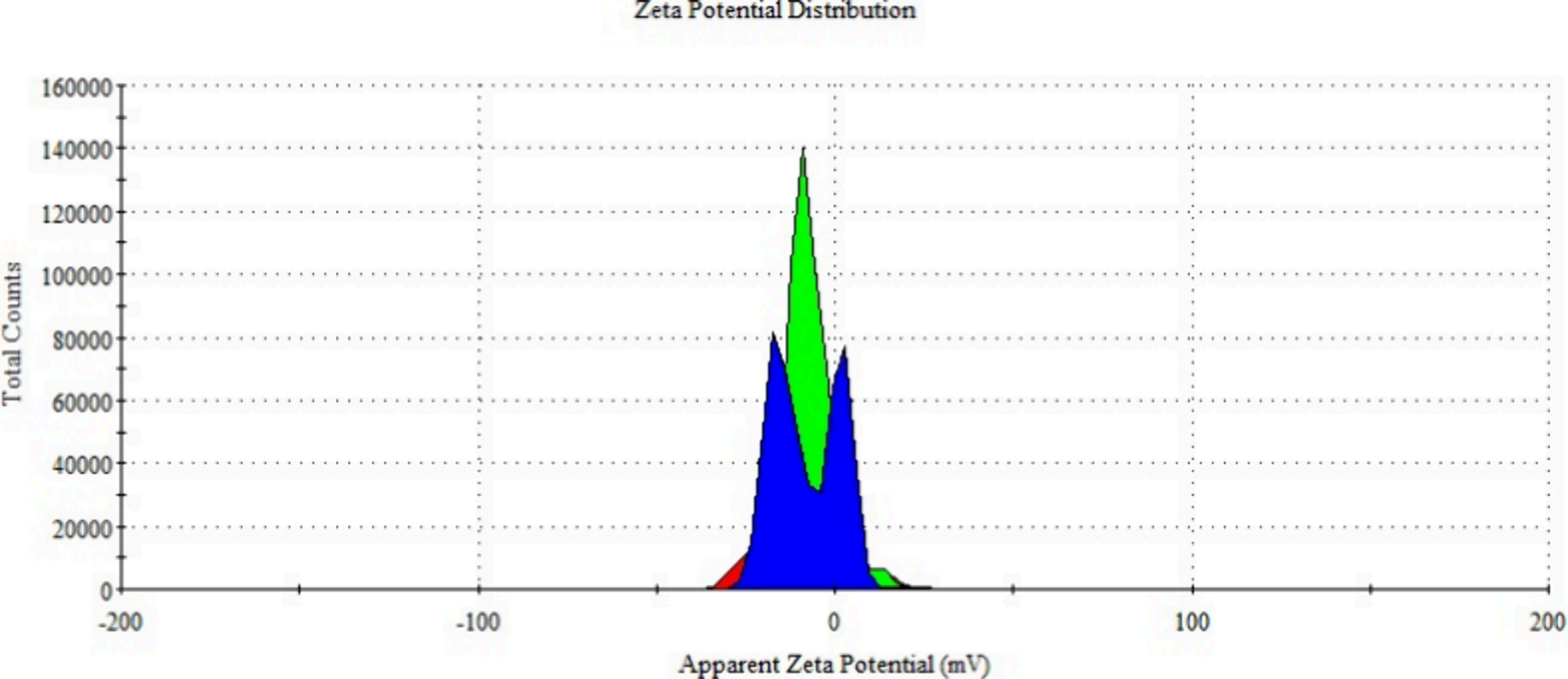
Zeta potential distribution of metformin-loaded niosome formulations.

### 3.2 Spectrophotometric Analysis

Regarding experimental findings, linearity for metformin was obtained in the 1–25 μg/mL concentration range with the regression coefficient (*R* ^2^) and the equation of the curve being 0.9994 and *y* = 0.0778*x* + 0.0548, respectively. The absorbance of each dissolution sample was measured at 232 nm, and concentration determinations were made in accordance with the equation (*y* = 0.0778*x* + 0.0548, *R* ^2^ = 0.9994) of the calibration curve.

### 3.3 The Drug Release Kinetic

The *in vitro* drug release profile of metformin HCl from the niosomal formulation was evaluated using the dialysis bag method in phosphate buffer (pH 7.4) at 37 °C. Cumulative release percentages were calculated by accounting for both the sampled and remaining drug amounts at each time point. As shown in Figure 13, a rapid initial release was observed during the first 120 min, which gradually plateaued over time, reaching approximately 100% release at 480 min. This biphasic pattern suggests an initial burst release of surface-associated drug, followed by a slower, diffusion-controlled release from the niosomal matrix.

**Figure 13:**
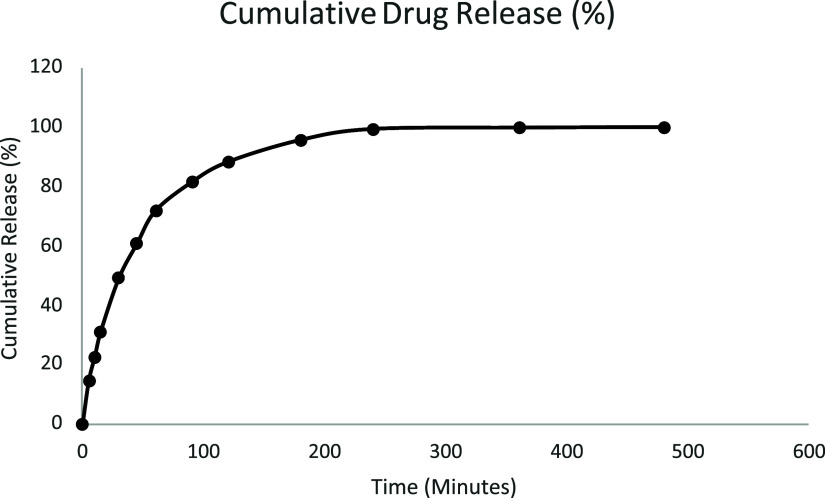
*In vitro* cumulative drug release profile of metformin-loaded niosomes over time.

The release data were fitted to multiple kinetic models to determine the release mechanism. In this graph, a linear regression applied to the zero-order model yielded an equation of *Y* = 0.1818*x* + 39.711 with a correlation coefficient of *R* ^2^ = 0.5904. The relatively low *R* ^2^ value indicates a poor fit to zero-order kinetics, suggesting that drug release was not constant over time and may instead be governed by diffusion-based mechanisms. Accordingly, better fitting was observed with the Korsmeyer–Peppas model (Table 2), supporting the hypothesis that the release was primarily controlled by diffusion through the vesicular matrix.

**Table 2:** Release Kinetics Parameters of Niosomal Formulations

kinetic model	correlation coefficient (*R* ^2^)	release constant
zero order	0.7779	*K*_0_ = 11.9741
first order	0.8484	*K*_1_ = −0.0846
Higuchi	0.9256	*K*_H_ = 29.9579
Hixson–Crowell	0.8258	*K*_HC_ = −0.2551
Korsmeyer–Peppas	0.9571	*N* = 0.5116

To gain deeper insight into the release mechanism of metformin from the niosomal formulation, the experimental data were fitted to various mathematical kinetic models. Among these models, the Korsmeyer–Peppas model provided the best fit to the experimental data with an *R* ^2^ value of 0.9571, indicating a strong linear correlation between log cumulative percent drug release and log time. Based on this model, the calculated *n* value was 0.5116, which falls within the range of 0.45 < *n* < 0.89, suggesting an anomalous (non-Fickian) transport mechanism. This indicates that the release process is governed by a combination of diffusion and swelling (polymer relaxation) mechanisms.

As shown in Figure 14, the practical release data (solid black line) closely follow the theoretical release profile (dotted blue line), further confirming the applicability of the Korsmeyer–Peppas model. Additionally, the relatively lower *R* ^2^ values observed for the zero-order (*R* ^2^ = 0.7779) and first-order (*R* ^2^ = 0.8484) models suggest that constant or concentration-dependent release mechanisms are less relevant for this formulation. The Higuchi model also demonstrated a good fit (*R* ^2^ = 0.9256), consistent with diffusion-controlled release, but the superior correlation of the Korsmeyer–Peppas model highlights its greater accuracy in describing the complex release behavior of metformin from niosomal systems.

**Figure 14:**
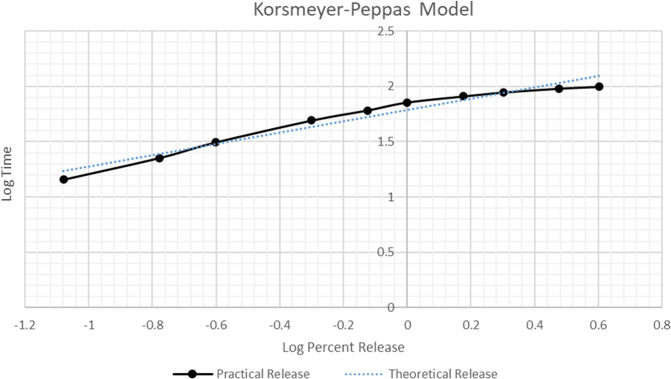
Korsmeyer–Peppas plot of log cumulative percent release versus log time for metformin HCl-loaded niosomes. The solid black line represents experimental (practical) release data, while the dotted blue line shows the theoretical linear fit. The release exponent *n* = 0.5116 suggests anomalous (non-Fickian) transport.

### 3.4 Measurement of Reactive Oxygen Species (ROS) Formation by Chemiluminescence

To compare the antioxidant activities of metformin-loaded niosomes with those of metformin treatment alone, ROS formation was assessed by the lucigenin–luminol chemiluminescence assay. While lucigenin specifically detects superoxide radicals, luminol measures other ROS such as OH^–^, H_2_O_2_, and HOCl.^37^ Based on our drug release analysis, isolated mouse brain tissues were incubated for 12 h with metformin alone, metformin-loaded niosomes, or null niosomes under equal conditions and concentrations. Pyro (0.1 mM, 5 min) significantly induced both superoxide radicals and other ROS formation in mouse brain compared to control (Figures 15 and 16, *p* < 0.001). Notably, Pyro significantly increased ROS formation in the null niosome group as well (Figures 15 and 16, *p* < 0.001). There was no statistical difference between Pyro or Pyro+null niosomes groups (Figures 15 and 16, *p* > 0.05). Treatment with metformin (1 mM, 12 h) or metformin-loaded niosomes (1 mM, 12 h) significantly reduced superoxide radicals (Figure 15; *p* < 0.01 and *p* < 0.001, respectively) and other ROS levels (Figure 16; *p* < 0.01 and *p* < 0.001, respectively). Importantly, metformin-loaded niosomes demonstrated a significantly greater reduction in both superoxide radicals and other ROS compared to metformin alone (Figures 15 and 16; *p* < 0.001 and *p* < 0.01, respectively), suggesting enhanced antioxidant efficacy of the niosomal formulation.

**Figure 15:**
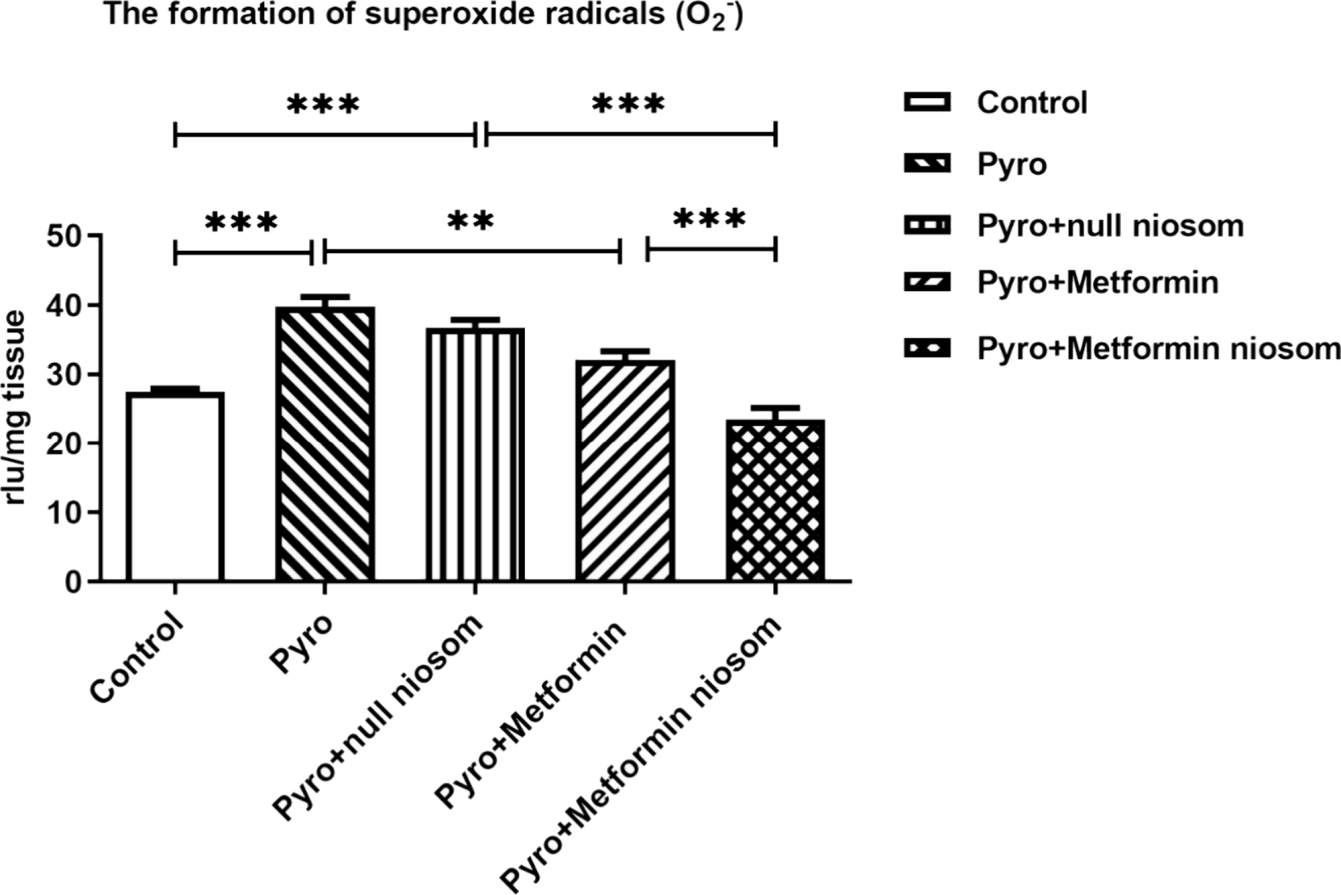
Effects of metformin alone (1 mM, 12 h) or metformin-loaded niosomes (1 mM, 12 h) on superoxide radicals induced by Pyrogallol (Pyro, 0.1 mM, 5 min) in the mouse brain. **p < 0.01, ***p < 0.001, One-way ANOVA with Bonferroni’s post hoc test, *n* = 5).

**Figure 16:**
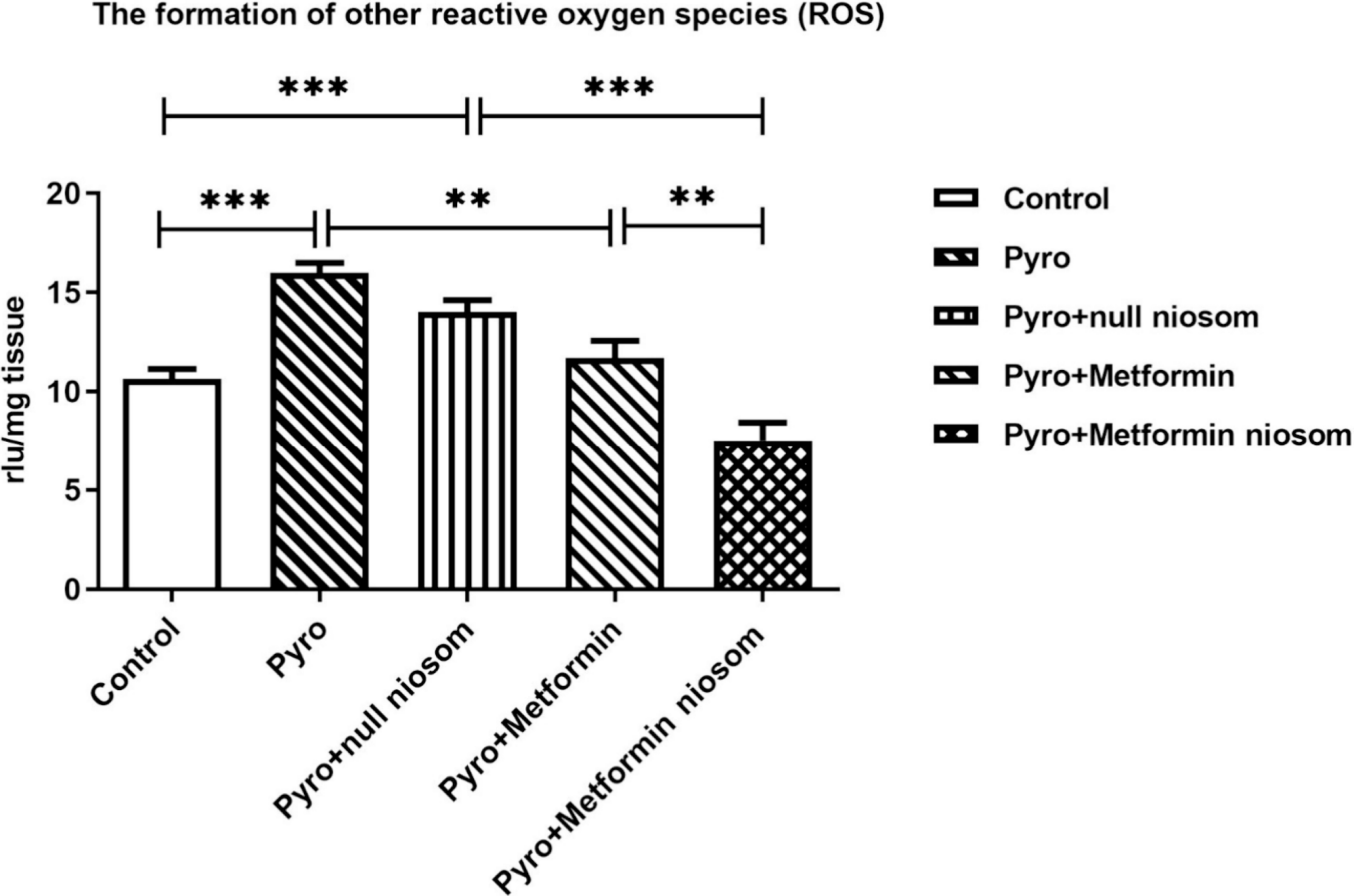
Effects of metformin alone or metformin-loaded niosomes on other ROS formation induced by Pyrogallol (Pyro, 0.1 mM, 5 min) in mouse brain. **p < 0.01, ***p < 0.001, One-way ANOVA with Bonferroni’s post hoc test, *n* = 5).

Brain tissue is particularly susceptible to free radical damage for various reasons (including high oxygen consumption, excessive amounts of rapidly peroxidable phospholipids, and the inability of neurons to regenerate). Disruption of the balance between oxidant and antioxidant systems in the organism in favor of oxidants causes the formation of various ROS such as superoxide (O_2_), hydrogen peroxide (H_2_O_2_), hypochlorite (OCl^–^), hydroxyl (OH^–^), and peroxide from molecular oxygen.^38^ These free radicals can react with many cellular molecules such as proteins, membrane lipids, and DNA and cause lipid peroxidation, protein oxidation, and DNA damage in cells.^39^ Such damage can disrupt cell membrane structure, impair cell functions, and even lead to cell death. In addition, increased ROS levels stimulate the release of proinflammatory cytokines and initiate inflammatory processes, thereby triggering brain inflammation.

T2DM has been suggested to be a risk factor for neurodegenerative diseases such as Alzheimer’s, Parkinson’s, and dementia. In recent years, preclinical and epidemiological studies have reported that T2DM is associated with disorders in memory and cognitive functions, and hyperglycemia causes neurodegeneration by increasing amyloid beta accumulation, oxidative stress, and neuroinflammation in brain.^40^ Therefore, the neuroprotective effects of metformin expand their therapeutic potential, particularly given the association of cognitive decline in diabetes and Alzheimer’s disease with type 3 diabetes mellitus (T3DM), often referred to as “brain diabetes”.^41^ We formulated metformin-loaded niosomes to enhance the brain delivery of metformin, taking advantage of their lipid bilayer and nanosized structure. The antioxidant activity of these niosomes was evaluated and compared with that of metformin treatment alone.

The superior antioxidant effect of metformin-loaded niosomes compared to metformin alone may be attributed to the niosomal system providing better drug penetration. The nanosized and bilayered structures of niosomes facilitate their intracellular entry and provide improved pharmacokinetic and delivery properties.^3^ Although we did not directly assess the pharmacokinetic and delivery properties in our study, niosomal formulation may enable greater intracellular uptake of metformin. Moreover, the sustained-release profile of metformin from niosomes may extend its bioavailability and intracellular retention, thereby enhancing its effectiveness in scavenging ROS. Furthermore, niosomal encapsulation may protect metformin from enzymatic degradation or rapid clearance, allowing for a more stable and efficient interaction with ROS-generating pathways.

In our study, we showed that metformin at 1 mM decreased Pyro-induced ROS formation in the brain. The concentration was chosen as the most effective concentration in studies showing the neuroprotective effects of metformin against oxidative stress induced by different oxidant agents.^42,43^ Katila et al. reported that 1 mM metformin was the concentration at which the most neuroprotective effect was observed against neuroblastoma cells against rotenone-induced oxidative damage.^12^ Although various preclinical studies have investigated the neuroprotective effects of metformin against different oxidant agents, its protective role against Pyro-induced oxidative stress has not been examined before. Pyro is known to induce oxidative stress and has been shown to reduce cell growth and induce apoptosis by elevating the O_2_^–^ radicals in various cells, including neuronal cells.^44^ Moreover, Pyrogallol is widely used in the hair dye industry in 1–5% (79–396 μM), which corresponds to the concentration that was used in our study. Thus, our study may have an importance in public health by showing antioxidant effects of metformin in detoxifying hair dye induced oxidative stress in brain.

Niosomes are drug delivery systems composed of nonionic surfactants, which have a lipophilic structure and increase the cross of drugs to the blood-brain barrier. We assume that the niosomes formulated in our study cross the BBB; however, this has not been experimentally confirmed, which constitutes a limitation of our work.

## Conclusions

In this study, metformin-loaded niosomes were formulated and characterized as a potential drug delivery system. The high encapsulation efficiency (68%) observed in the niosome formulations confirms the effectiveness of the selected synthesis method, particularly in achieving an optimal drug loading capacity. The particle size and polydispersity index values suggest that these niosomes are suitable for targeting tissues with high blood flow such as the brain, which is critical for their potential neuroprotective applications. The zeta potential measurements indicated the aim for aggregation, pointing to the necessity for additional optimization to enhance stability. Furthermore, in vitro dissolution studies revealed that the drug release from niosome formulation suits the Korsmeyer–Peppas kinetic model. Additionally, optimization studies should be carried out to increase the stability and therapeutic effectiveness of the formulation. Metformin-loaded niosomes significantly reduced ROS formation compared with metformin alone, suggesting enhanced neuroprotective effects. Overall, the results indicate that niosomes present a potential strategy for the targeted delivery of metformin, particularly for neurodegenerative diseases. In further experiments, the neuroprotective effects of metformin-loaded niosome formulations can be compared to those of metformin administered alone by establishing in vivo models that combine T2DM and neurodegenerative diseases.

## Data Availability

The data underlying this study are available in the published article. Further inquiries can be directed to the corresponding author.
